# Fast spectrally encoded Mueller optical scanning microscopy

**DOI:** 10.1038/s41598-019-40467-z

**Published:** 2019-03-08

**Authors:** Sylvain Rivet, Matthieu Dubreuil, Adrian Bradu, Yann Le Grand

**Affiliations:** 1 Laboratoire d’Optique et de Magnétisme, Université de Bretagne Occidentale, IBSAM, 6 avenue Le Gorgeu, 29238 Brest, France; 2 Applied Optics Group, School of Physical Sciences, University of Kent, Canterbury, CT2 7NH UK

## Abstract

Mueller microscopes enable imaging of the optical anisotropic properties of biological or non-biological samples, in phase and amplitude, at sub-micrometre scale. However, the development of Mueller microscopes poses an instrumental challenge: the production of polarimetric parameters must be sufficiently quick to ensure fast imaging, so that the evolution of these parameters can be visualised in real-time, allowing the operator to adjust the microscope while constantly monitoring them. In this report, a full Mueller scanning microscope based on spectral encoding of polarization is presented. The spectrum, collected every 10 μs for each position of the optical beam on the specimen, incorporates all the information needed to produce the full Mueller matrix, which allows simultaneous display of all the polarimetric parameters, at the unequalled rate of 1.5 Hz (for an image of 256 × 256 pixels). The design of the optical blocks allows for the real-time display of linear birefringent images which serve as guidance for the operator. In addition, the instrument has the capability to easily switch its functionality from a Mueller to a Second Harmonic Generation (SHG) microscope, providing a pixel-to-pixel matching of the images produced by the two modalities. The device performance is illustrated by imaging various unstained biological specimens.

## Introduction

A key method in biological research, light microscopy has, for centuries, been looking for new approaches to enhance contrast by making use of the wave nature of light, the linear and/or non linear optical properties of matter and the addition of specific chromophores. Among numerous optical microscopy imaging modalities, polarized light microscopy^1^ is dedicated to the observation of biological structures^2^ that exhibit intrinsic optical anisotropic properties in phase (linear/circular birefringence) and amplitude (linear/circular diattenuation) at sub-micrometer scale. The main source of anisotropy in biological tissues, especially those of mammals, is linear birefringence caused by a few regular arrangements of lipids or proteins such as collagen and elastin fibres in connective tissues, microtubules and actin filaments in cytoskeleton, myelin sheath in brain tissue, amyloid fibrils related to Alzheimer’s and prion diseases. Plants also exhibit linear birefringence through structural (cellulose fibres) and storage (semi-crystalline starch) polysaccharide building blocks. Linear diattenuation of biological structures could originate from reflections by the curvature of the specimen surface through Fresnel’s laws, from scattering by particles (Rayleigh-Gans theory), but also from absorption associated to anisotropic transition moments. In the latter case, diattenuation is often called dichroism and it was, for instance, experimentally assessed in brain nerve fibres^3^. Circular birefringence (also called optical rotation) and circular diattenuation (also called circular dichroism) originates from chiral molecules and/or structures. In biological fluids or tissues, optical rotation is mainly significant in the presence of glucose and it was demonstrated that circular dichroism is for example sensitive to the structure of chromatin in cells^4^. Finally, due to multiple scattering on various centres through specimens, light polarization can also be randomly modified (depolarization), which cannot be circumvented in thick biological tissues. For the last decades, spatial mapping at microscopic scale of polarization-based optical effects was demonstrated to be a powerful label-free method to analyse the architecture of tissues and their alteration due to different kinds of pathologies.

The usual white light polarization microscope, in which the sample is placed between crossed polarizers, is routinely used for qualitative imaging but can sometimes provide ambiguous image contrasts in cases where several effects occur simultaneously. To obtain quantitative information and automate the measurement process, other schemes have been proposed involving compensators^5^, rotating optical components^6^ or electro-optic modulators^7,8^ to give only a few examples. Except Stokes microscopes^9^ that measure degree of polarization, most of them assumed that the specimens exhibit a pure polarimetric effect, *i*.*e*. linear birefringence, circular birefringence, linear diattenuation or circular diattenuation. As the need has arisen to take mixed polarimetric effects into account, confocal scanning Mueller microscopes^10,11^ and full-field transmission Mueller polarimetric microscopes have been successfully devised and then used for imaging biological tissues^12–17^. The full-field microscopes have been designed with incoherent light sources and CCD or CMOS sensors in various ways, from dual rotating compensators^18^ to liquid crystal variable retarders^19^, using different experimental strategies to measure the full Mueller matrix^20–23^ containing all the polarimetric features of specimens. Benefiting from the advances in Mueller matrix decomposition^24,25^, Mueller microscopes have the great advantage of providing all the elements that will produce multiple images resulting from the exhaustive polarimetric response of the specimen, such as linear retardance *R*_*L*_ (associated to linear birefringence) and the orientation (or azimuth) of its slow axis *α*_*R*_, linear diattenuation *D*_*L*_ and the orientation (or azimuth) of its axis α_*D*_, circular retardance *R*_*C*_ (associated to circular birefringence), circular diattenuation *D*_*C*_, depolarization parameter^26^ Δ. Nevertheless, such microscopes still have limited performance due to: (i) the complexity of the polarization state generation and analysis and (ii) the time delay, typically from a few dozens of seconds to few minutes, for recording enough images to retrieve the 16-elements of the Mueller matrix with a suitable signal-to-noise ratio. Another limitation, usually not mentioned in most studies, is the difficulty of displaying these polarimetric parameters in real time, which is a huge issue when the operator adjusts the microscope to find a region of interest. Indeed, in addition to acquisition time, a long post-processing stage is necessary to compute the Mueller matrices, to correct numerically the polarimetric signature of the microscope itself and to interpret Mueller matrices physically. Due to these limitations, Mueller microscopy is not widespread yet despite its interesting use in imaging histological sections of liver collagen fibers^27,28^ stained with picro-sirius red to increase birefringence, Morpho butterfly^29^ or crystals^30^ for instance.

Recently, we have demonstrated as a proof of concept, the first full Mueller polarimetric scanning microscope based on an optical frequency swept-laser source implemented within a commercial confocal-like microscope^31^. The device did not use any moving parts for the polarized state generator (PSG) and analyser (PSA) blocks, but only static polarizers and retarders to spectrally encode light polarization^32–34^. From the channelled spectrum measured by a single-pixel detector, the full Mueller matrix was determined at each point of the specimen. The tuning speed of the swept-source laser, (10 μs in our case) determined the data acquisition time of our device. That enhanced microscope benefited from a simpler design due to the use of passive PSG and PSA blocks, and significantly improved signal-to-noise ratio as a result of the coherent point-to-point illumination.

This report presents a full Mueller scanning microscope based on new designs of passive PSA and PSG blocks in order to display in real-time linear birefringence and its orientation or more generally the change of polarization induced by the specimen as guidance for the operator whilst adjusting the microscope. In addition, the instrument is provided with another functionality allowing the operator to switch easily from a Mueller to a Second Harmonic Generation (SHG) imaging modality, while keeping a perfect point-to-point matching of the images. The performance of our new Mueller microscope is illustrated through imaging various unstained biological specimens such as potato starch granules, aponeurosis in chicken legs and human liver with fibrosis disease, in order to show the ability to separate different anisotropies within specimens. All the polarimetric features are recorded at frame rates of 1.5 Hz and 0.4 Hz from respectively single-scan 256 × 256 and 512 × 512 images (to our knowledge, this is the highest rate ever reported). Firstly, a video showing potato starch granules has been produced during the adjustment of the microscope, to demonstrate the possibility of real-time imaging of linear birefringence (amplitude and axis orientation) as guidance. Secondly the images of retardance, diattenuation and depolarization of the same potato starch granules have been made through a Mueller analysis. Thirdly another video shows aponeurosis being altered by hydrochloric acid to demonstrate the ability of the instrument to image simultaneously all the polarimetric effects at high speed. Finally, the same liver specimen has been scanned using both Mueller and SHG imaging pathways of our microscope. This illustrates how a well-designed Mueller modality is able to reveal unstained birefringent arrangements – such as fibrillar collagen within thin tissue sections – and competes with a much more expensive nonlinear SHG modality.

## Results

### Real-time image display

**Figure 1:**
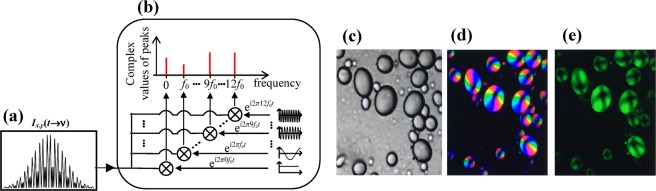
Signal processing for real-time display. (**a**) Channelled spectrum measured versus time by the detector for the Mueller microscopy modality, originating from a single point on the specimen. (**b**) Extraction of the signal modulations by local oscillators. (**c**) Grey level image of the DC component. (**d**) HSV image of the 9^th^ peak. Hue determined by the phase of the peak and brightness by its amplitude. **(e)** Green level image of all the modulations ∑_k_ _=__1,12_ [|Δ*P*_*k*_|] except the DC component. Images (**c**–**e**) without any average.

The measurement of the Mueller matrix of a specimen, allowing access to its complete polarimetric information, requires generating several input polarization states and analysing these states modified by the specimen. Instead of carrying out the measurements by active PSG and PSA blocks using rotating optical elements or polarization modulators, PSG and PSA blocks are made of thick chromatically dispersed retarders associating each polarization state to a wavelength and permitting to perform both generation and analysing in a passive way. The microscope is based on a standard upright confocal microscope with these PSG and PSA blocks respectively at the input and the output of the microscope in such a way that polarization states are generated and analysed for each wavelength delivered in time by the swept-source laser. The channelled spectrum *I*_*x*,*y*_(*t*) measured in transmission (Fig. 1(a)) is the combination of 12 modulations from *f*_0_ to 12 *f*_0_ whose amplitudes and phases depend on the elements *m*_*ij*_ of the Mueller matrix related to the polarimetric signature at each point of the specimen, the microscope itself and the PSG and PSA blocks. The acquisition of the spectrum takes 10 μs per image pixel, according to the tuning speed of the swept-source used in these experiments, which allows for producing the Mueller components at the usual frame rate of optical scanning microscopes, once raw data are processed. To obtain images resulting from the polarimetric signature of the specimen only, several steps of post-processing detailed in the “Methods” section are carried out, which we refer to as the Mueller analysis. However, all the steps, from Fourier transformation – measuring the amplitude and phase of modulations – through the numerical correction of the polarimetric response of the microscope, to matrix decomposition, increase the processing time, and do not allow for displaying in real-time all the polarimetric images of the specimen. Instead of speeding up calculations by implementing the post-processing algorithms on FPGA or GPU, our approach was to display only specific images for guidance for the microscope operator through a software developed in LabVIEW 2017 (National Instruments, Austin, Texas). For this purpose, three modifications have been made to our Mueller microscope. First a Berek compensator was added to compensate at least for the linear birefringence induced by the fold mirrors used to convey light from the swept-source laser into the microscope and by the galvo-scanner mirrors. Second, one achromatic quarter-wave plate has been added to the initial PSG as well as PSA blocks so that particular modulations of the channelled spectrum *I*_*x*,*y*_(*t*) are directly linked to linear birefringence as described in the “Methods” section. Third, the amplitude and the phase of the modulations of *I*_*x*,*y*_(*t*) are measured with numerical oscillators^35^ instead of using a Fourier transform. This strategy is more efficient to tackle real-time issues, as the axial range of interest is shorter than the one obtained via Fast Fourier Transform^36^. To measure the complex values of the peaks *P*_*k*_ associated to the 12 modulations (*k* = 0 to 12), *I*_*x*,*y*_(*t*) is only multiplied with 12 numerical oscillators $Exp(i\,2\pi \,k\,{f}_{0}\,t)$, and the difference between the peaks Δ*P*_*k*_ with and without the specimen is calculated. Note that the peaks *P*_*k*_ (for *k* ≠ 0) are normalized according to the DC component *P*_0_ in order to remove the transmittance of the specimen. Figure 1(c–e) are images of a potato starch granule specimen obtained by displaying different peaks Δ*P*_*k*_. When observed under polarized light, starch granules exhibit a Maltese cross that indicates a radial orientation of the principle axis of birefringence, while retardance remains almost unchanged. Further details about starch granules will be given in the next section. Figure 1(c) corresponds to the peak Δ*P*_0_ in grey levels that is mainly sensitive to the transmittance of the sample. Figure 1(d) is an image for which the phase of the peak Δ*P*_9_ is colour-coded in Hue-Saturation-Value (HSV) space, while the brightness of colours is regulated by the amplitude of Δ*P*_9_, the saturation being set to 1. The peak Δ*P*_9_ has been chosen because it theoretically depends on the linear retardance *R*_*L(x*,*y)*_ and its azimuth α_*R(x*,*y)*_ through the expression ${\rm{\Delta }}{P}_{9}=2\,\sin \,{R}_{L(x,y)}\,Exp(-i\,2\,{\alpha }_{R(x,y)})$, supposing that the anisotropy of the microscope has been compensated for. Thus, unlike Fig. 1(c,d) shows the birefringence of the starch granule and the rotation of its azimuth around a point whose retardance is zero. Figure 1(e) corresponds to ∑_k=1,12_ [|Δ*P*_*k*_|] in green levels that images the starch granule on a dark background and is sensitive to an overall change of polarization. This parameter is less sensitive to the orientation of the birefringence and exhibits a low contrasted Maltese cross. A recording of the computer screen that displays in real-time the image of the specimen during the microscope adjustments can be found as Supplementary Video S1. It demonstrates the possibility of switching between Δ*P*_9_ or ∑_*k*=1,12_ [|Δ*P*_*k*_|], zooming in and zooming out, and moving the specimen laterally. Peaks ΔP_3_, ΔP_7_ and ΔP_11_ are similar to ΔP_9_ and could have been used in the same way. As the other peaks depend on both birefringence and diattenuation, it is not possible to directly display the diattenuation unless the sample is a pure diattenuator. Figure 1(c–e) can be used conveniently as guidance for the specimen settings under the microscope objective. They cannot however replace images from the Mueller analysis to obtain truly quantitative information on the specimen.

### Imaging potato starch granule through the Mueller analysis

**Figure 2:**
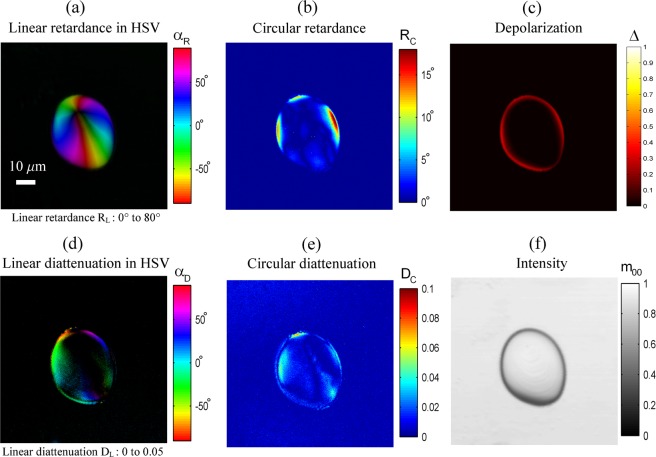
Images of a potato starch granule. Images of size 90 × 90 µm (512 × 512 pixels) without any average. Microscope objective: 20X, NA = 0.75. (**a**) Linear retardance image in HSV (Hue = azimuth of retardance, Saturation = 1, Value = linear retardance). (**b**) Circular retardance image in false colour. (**c**) Depolarization image. (**d**) Linear diattenuation image in HSV (Hue = azimuth of diattenuation, Saturation = 1, Value = linear diattenuation). (**e**) Circular diattenuation image in false colour. **(f)** Intensity image (*m*_00_) in gray level.

Starch granules^37^ are excellent specimens for highlighting the sensitivity of the swept source Mueller microscope. Starch is produced by green plants for energy storage over long periods of time and is mainly found in seeds, roots and tubers. Starch grains grow from a central point, the hilum, with growth rings alternating amorphous (amylose) and semi-crystalline line shells (amylopectin). The granules occur in all shapes and sizes (spheres, ellipsoids, polygons, irregular tubules, etc) according to their botanical origin. Figure 2 shows an image of a starch granule produced by our Mueller microscope through a Mueller analysis after Lu and Chipman matrix decomposition. Axis orientation values α_*R*_ of linear retardance are colour-coded in Hue-Saturation-Value (HSV) space, while the brightness of colours is regulated by the retardance values *R*_*L*_, the saturation being fixed to 1. The same procedure has been used for the linear diattenuation with the value corresponding to diattenuation *D*_*L*_ and hue representing its azimuth α_*D*_. Thus, Fig. 2 contains 6 images resulting from the cumulative effect of polarization modifications along the propagation of light in the specimen. Predictably, as shown in Fig. 2(a), the starch granule is mainly equivalent to a linear retarder whose azimuth rotates around the hilum, as the linear retardance reaches a maximum of 80°. Nevertheless, other polarimetric effects appear near the boundaries of the granule but also within the granule itself. This requires the use of the Mueller analysis to separate the effects without corrupting the linear retardance measurement. In particular, depolarization (Fig. 2(c)) is visible at the edges of the granule. The presence of circular retardance (Fig. 2(b)) superimposed with linear retardance indicates that the specimen is equivalent to an elliptical birefringent. Whereas circular retardance is weak, it is beyond the background noise of the Mueller microscope (evaluated at a later stage with reference samples). Ellipticity could be a result of the sample’s inhomogeneities from a distribution of the orientation of the optical retardance axes within the granule volume. Linear and circular diattenuations (Fig. 2(d,e)) also appear on the periphery of the granule and to a smaller extent on the inside. The origin of this effect is not clear and it could originate from the anisotropic Fresnel coefficients describing the transmission of the *s* and *p* polarizations incident upon the surfaces of the granule. The ellipticity of the diattenuation could be explained by a misalignment of the principal planes of the input and output surfaces with respect to the optical beam. Another explanation for diattenuation could be a possible anisotropic absorption of amylopectin, but this hypothesis is less likely because to our knowledge, there is no mention in literature of any significant dichroism of amylopectin at 1050 nm. Moreover, the images of retardance and diattenuation should be identical if they have a common origin (amylopectin), which is not the case when circular retardance and circular diattenuation are compared.

### Imaging degradation of aponeurosis in chicken leg tissues

**Figure 3:**
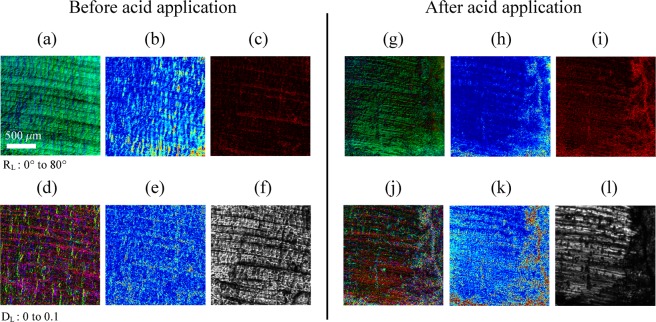
Polarimetric images of aponeurosis in chicken legs. Images of size 1.7 × 1.7 mm (256 × 256 pixels) without any average. Microscope objective: 4X, NA = 0.16. Before and one minute after contact with acid, (**a**,**g**) linear retardance image in HSV (Hue = azimuth of retardance, Saturation = 1, Value = linear retardance), (**b**,**h**) circular retardance image in false colour, (**c**,**i**) depolarization image, (**d**,**j**) linear diattenuation image in HSV (Hue = azimuth of diattenuation, Saturation = 1, Value = linear diattenuation), (**e**,**k**) circular diattenuation image in false colour, (**f**,**l**) intensity (*m*_00_) image in gray level.

Aponeurosis is a dense regular connective tissue which attaches muscles to bones. Structurally aponeuroses are flat sheets of densely oriented collagen fibres and generally consist of several layers of different orientations. As a result, aponeuroses can expand both parallel and perpendicular to a muscle’s line of action^38^. Figure 3 shows the images of the intramuscular aponeurosis of a chicken leg tissue, produced according to the same modality as in the last section, *i*.*e*. 6 images corresponding to linear retardance in HSV, circular retardance, linear diattenuation in HSV, circular diattenuation, depolarization and transmitted intensity. These images are obtained before and one minute after hydrochloric acid application. Before application, two layers of oriented collagen fibres can be seen through the linear retardance (Fig. 3(a)) and the circular retardance (Fig. 3(b)). In fact, the addition of two linear birefringent samples whose axes are not aligned is equivalent to an elliptical retardance that can be separated into linear and circular retardance. The meshing resulting from the superposition of layers is also highlighted in the linear diattenuation image (Fig. 3(d)). When acid is spread on the surface of the specimen, the collagen is denatured hence a decrease of the birefringence strength. This alteration is characterized by a drop of the value in the HSV image in Fig. 3(g) and the loss of circular birefringence structure in Fig. 3(h) suggests that one of the layers is totally altered. The polarimetric changes of the aponeurosis caused by acid have been recorded at the frame rate of 1.5 Hz and displayed 10 times as fast in the Supplementary Video S2.

### Imaging unstained liver fibres fixed in paraffin

**Figure 4:**
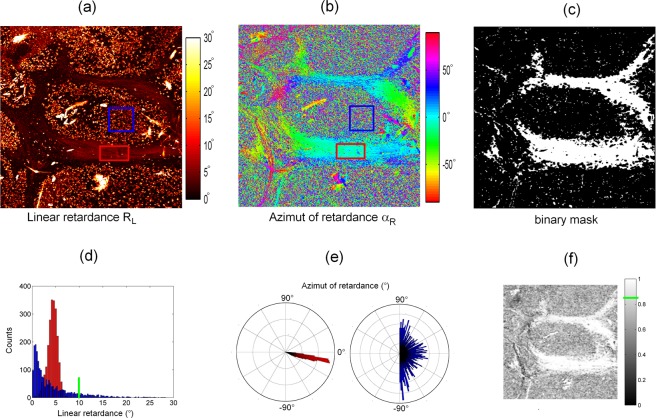
Linear retardance images of a liver specimen and creation of a binary mask based on the degree of alignment (DoA) of retardance. Images (**a**,**b**) of size 1.7 × 1.7 mm (256 × 256 pixels) without any average. Microscope objective: 4X, NA = 0.16. (**a**) Linear retardance image. (**b**) Azimuth of retardance image. (**c**) Binary mask calculated from the thresholds defined by the green line on the grayscale bar of (**f**) and on the linear retardance histogram of (**d**). (**d**) Histograms of the linear retardance calculated in the red and the blue regions defined in (**a**). The red region mainly contains collagen fibres whereas the blue region mainly contains paraffin. (**e**) Histograms of retardance azimuth calculated in the same red and blue regions. (**f**) DoA image of the specimen.

An unstained liver specimen at F4 fibrosis stage (see “Methods section) was imaged by our microscope. It is well established that liver fibrosis is characterized by an over-production of fibrillar collagen as the disease progresses^39^. Figure 4(a,b) display respectively linear retardance and azimuth of retardance images. The specimen mainly exhibits linear birefringence, as expected for fibrillar collagen of type I^40^. All other polarimetric effects are weak at the locations of collagen within the specimen, and were thus not presented in this paper. It can be noticed that the linear retardance image is polluted by a strong background signal. This polarimetric signature can be attributed to paraffin that has not been correctly removed from the preparation as paraffin retardance values (up to 30°) are far above those of collagen fibres themselves (around 5°). Collagen fibres then clearly appear in negative contrast in Fig. 4(a). We have analysed the behaviour of the linear retardance and the azimuth of retardance within selected areas mainly constituted of collagen fibres and paraffin (respectively the red and blue boxes defined in Fig. 4(a,b)).

**Figure 5:**
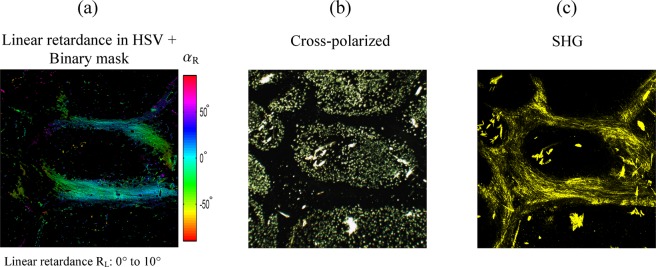
Comparison between Mueller, cross-polarized and SHG images of the liver specimen. (**a**) Linear retardance image in HSV (Hue = azimuth of retardance, Saturation = 1, Value = linear retardance). Same acquisition as in Fig. 3. (**b**) Same area imaged with a wide field polarized microscope (crossed polarizers). (**c**) Same area imaged in second harmonic generation (SHG). “Hot regions” in (**b**) correspond to a mixing of linear retarders with different orientations and cannot be eliminated between a rotated pair of crossed polarizers.

One can notice in Fig. 4(d) that the distribution of the linear retardance values in the paraffin area is wide (from 0° to 30°), preventing us from applying a numerical threshold method based on linear retardance value to remove paraffin from the images. However, Fig. 4(e) shows that the collagen area (red plot) is characterized by a narrow orientation distribution of birefringence axes unlike regions containing paraffin (blue plot). In order to remove paraffin from polarimetric images, we therefore defined a degree of alignment (DoA) of birefringence axes written as DoA = (<sin β>^2^ + <cos β>^2^)^1/2^ where < > is the mean value and β is the set of the considered birefringence azimuths. DoA values range from 0 for totally disordered to 1 for perfectly aligned azimuths of retardance between neighbouring pixels. Figure 4(f) shows the corresponding image of DoA applied to all the specimen, the averaged DoA values being equal to 0.72 and 0.95 for the region containing respectively paraffin and collagen fibres. From thresholds based first on the DoA image and secondly on the linear retardance image as evidenced by the green lines in Fig. 4(d,f), a binary mask (Fig. 4(c)) was then created and applied to the linear retardance image leading to Fig. 5(a).

In Fig. 5(a), most of the paraffin signature was removed by implementing the numerical method based on DoA filtering. Collagen fibres are now clearly visible as well as their orientation. To demonstrate the capability of the Mueller polarimetric microscope to image unstained liver biopsy embedded in paraffin, the same area was imaged using a standard transmission polarization microscope. The cross-polarized image is represented in Fig. 5(b). This image mainly reveals paraffin that is strongly birefringent and thus appears in white in the cross-polarized image. Collagen fibres have a much lower birefringence and are invisible in this image. Moreover, in a classical polarization microscope, the contrast of collagen fibres depends on the alignment with directions of the polarizers, and vanishes for a perfect alignment. Collagen fibres can be made visible by rotating the specimen. This makes the use of a standard polarization microscope for visualization of unstained liver biopsy embedded in paraffin very difficult unlike Mueller microscopes whose contrast is independent of the orientation of the specimen. In addition, a numerical method based on the azimuth of retardance enables to remove highly dispersed paraffin from the retardance image. The same region was imaged by switching the microscope to the SHG configuration (Fig. 5(c)). The SHG image specifically reveals type-I and III fibrillar collagen and was demonstrated to be a relevant tool for assessing liver fibrosis^41,42^ but its nonlinearity complicates polarization parameter extraction and interpretation^43,44^. One should note that residual paraffin is visible on the SHG image, corresponding to the “hot” regions in the cross-polarized image (Fig. 5(b)). This indicates a higher organization of paraffin at the scale of the focal excitation volume of the microscope. The HSV linear retardance and SHG images are very close, confirming that the retardance is indeed produced by fibrillar collagen. The pixel-to-pixel comparison of the two images has not been fully exploited in this paper and further studies will be dedicated to a thorough analysis of this comparison, adding a polarization-resolved modality for SHG to reach full linear/nonlinear polarimetric characterization. Note that specimens have seldom been imaged with the double Mueller/SHG modality of the same scanning microscope and have only been proposed recently in reflection^45^.

## Discussion

We have developed a multi-modal optical scanning microscope able to (i) display in real-time birefringent images coded in HSV space where hue is generated for any specimen orientation, (ii) measure the full Mueller matrix at each point on the specimen at the typical frame rate of optical scanning microscopes, (iii) produce SHG images with the same pixel-to-pixel matching as Mueller images. Our full Mueller scanning microscope benefits from the latest advances in Optical Coherent Tomography^46^ (OCT) technology in terms of light source, digital acquisition card and post-processing methods. Indeed, OCT and our Mueller microscope are both optical scanning methods and all the relevant information is contained in the modulations of the channelled spectra (the depth-resolved reflectivity profile for OCT, the elements of the Mueller matrix for the Mueller microscope). So far, swept-sources have been developed in the near-IR band, which can be a limitation if we are interested in the measurement of dichroism or depolarization related to absorption and/or scattering properties which are usually stronger in the visible band. Nevertheless, OCT technologies are continually evolving by increasing the speed of the swept-sources^47^ and developing new swept-sources in the visible range^48^, which could be an asset for future innovations in Mueller microscopy. To prevent negative impact of high NA on polarimetric parameters^49^, all images were produced using with microscope objectives whose NA is lower than 0.8, which limits the spatial resolution of the images. In Mueller microscopes, like in all polarized microscopes, a compromise between spatial resolution and precision of the polarimetric measurement must be made.

## Methods

### Experimental setup

#### Mueller polarization modality

**Figure 6:**
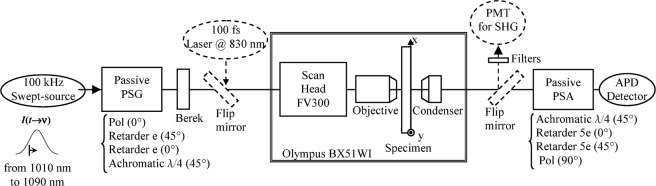
Experimental setup. Description of the experimental setup based on a swept-source for the Mueller microscopy modality and on a femtosecond laser for the Second Harmonic Generation modality.

The swept-source, (SSOCT-1060, Axsun Tech. Inc.) sweeps the optical frequency over a spectral range of 100 nm (before digitization) around 1050 nm at a rate of 100 kHz. Figure 6 shows the PSG and PSA blocks. They incorporate: a linear polarizer (Pol) oriented at 0° or 90°, YVO4 thick retarders oriented at 45° and 0°, achromatic quarter wave-plates at 45°, all the optical elements in the PSG and PSA being fixed. The YVO4 thickness is *e* = 0.4 mm for PSG and 5*e* = 2 mm for PSA. The electrical signal delivered by an avalanched photodiode (APD module C12703SPL, Hamamatsu) equipping a standard upright confocal microscope (Olympus BX51W1-FV300) is digitized by a data acquisition board (DAQ, ATS9350 digitizer, AlazarTech) in synchronism with the galvo-scanners the microscope is equipped with. Technical details of synchronization between the swept-source, galvo-scanners and DAQ have been disclosed in ref.^31^. The digitized signal includes a series of channelled spectra whose modulation is related to the polarimetric signature of each pixel of the image with a pixel-dwell time equal to 10 μs.

#### SHG modality

The femtosecond laser is a Coherent Mira-Verdi femtosecond laser tuned at 830 nm and the SHG signal is detected through a 415 nm filter by a photomultiplier module working in transmission. Flip mirrors have been added in the optical path to inject a femtosecond laser to switch from one imaging modality to the other. Using the same microscope for both Mueller matrix measurement and SHG detection allows a pixel-to-pixel comparison of the images while the usual practice consists in imaging specimen with two different devices^50,51^.

#### Addition of achromatic quarter wave plates

In the basic configuration of PSG and PSA blocks described in ref.^33^, each channelled spectrum *I*_*x*,*y*_(*t*) obtained at the coordinate (*x*, *y*) in the sample is a multi-periodic signal corresponding to the first component of the output Stokes vector ${\overrightarrow{S}}_{o}$ defined in the Stokes-Mueller formalism by

1 $${\overrightarrow{S}}_{o}=[{M}_{PSA(5{\rm{\Phi }})}]\cdot [M]\cdot [{M}_{PSG({\rm{\Phi }})}]\cdot {\overrightarrow{S}}_{i},$$

2 $${\rm{\Phi }}=\frac{2\pi \,{\rm{\Delta }}n\,e}{c}{\nu }_{(t)}\approx {{\rm{\Phi }}}_{0}+{f}_{0}\,t\,$$

3 $$[M]=[\begin{array}{cccc}{m}_{00} & {m}_{01} & {m}_{02} & {m}_{03}\\ {m}_{10} & {m}_{11} & {m}_{12} & {m}_{13}\\ {m}_{20} & {m}_{21} & {m}_{22} & {m}_{23}\\ {m}_{30} & {m}_{31} & {m}_{32} & {m}_{33}\end{array}],$$

where [*M*], [*M*_*PSG*_] and [*M*_*PSA*_] are respectively the Mueller matrices of the unknown sample, the PSG and PSA blocks, the optical frequency *v*(*t*) is delivered in time by the swept-source, Δ*n* is the birefringence of the YVO4 retarders, ${\overrightarrow{S}}_{i}$ is the input Stokes vector. With the addition of achromatic quarter wave-plates to devise the new PSG and PSA blocks, the relationships between the modulations of *I*_*x*,*y*_(*t*) and *m*_*ij*_ elements can be inferred by noting that Eq. (1) can be written as follows with the initial PSG and PSA blocks, $[{M}_{PSG}^{ref\,[33]}]$ and $[{M}_{PSA}^{ref\,[33]}]$ defined in ref.^33^:

4 $${\overrightarrow{S}}_{o}=[{M}_{PSA\,(5{\rm{\Phi }})}^{ref\,[33]}]\cdot [M^{\prime} ]\cdot [{M}_{PSG\,({\rm{\Phi }})}^{ref\,[33]}]\cdot {\overrightarrow{S}}_{i},$$

5 $$[{M}^{{\rm{^{\prime} }}}]=[{M}_{{45}^{{}^{\circ }}}^{\lambda /4}]\cdot [M]\cdot [{M}_{{45}^{{}^{\circ }}}^{\lambda /4}]=[\begin{array}{cccc}{m}_{00} & -{m}_{03} & {m}_{02} & {m}_{01}\\ {m}_{30} & -{m}_{33} & {m}_{32} & {m}_{31}\\ {m}_{20} & -{m}_{23} & {m}_{22} & {m}_{21}\\ -{m}_{10} & {m}_{13} & -{m}_{12} & -{m}_{11}\end{array}],$$

with $[{M}_{{45}^{^\circ }}^{\lambda /4}]$ the Mueller matrix of an achromatic quarter-wave plate oriented at 45°. In particular the 9^th^ peak ΔP_9_ can be expressed as ${\rm{\Delta }}{P}_{9}={e}^{2i\varepsilon }(2\,{m^{\prime} }_{21}-2i\,{m^{\prime} }_{31})={e}^{2i\varepsilon }(-2\,{m}_{23}-2i\,{m}_{13})$ where ε is a constant coming from the thickness of YVO_4_ retarders that are not perfectly equal to *e* or 5*e*. For a specimen that does not exhibit any polarimetric effects or only diattenuation or circular birefringence, ΔP_9_ is zero. On the other hand for a pure linear birefringence, we have ${\rm{\Delta }}{P}_{9}=2\,\sin \,{R}_{L(x,y)}\,Exp(-i\,2({\alpha }_{R(x,y)}+\varepsilon ))$, thus the amplitude of the peak is related to retardance and the phase to the orientation of its axis, facilitating real-time display of birefringence. Note that the addition of achromatic quarter-wave plates do not change the equally weighted variance^52,53^ (EWV) of the device (figure of merit quantifying the well-conditioning of the measurement) because they do not participate to the coding/decoding of the polarization states since they are achromatic while the coding/decoding is based on the chromatic dispersion of the optical elements.

#### Mueller analysis

Signal post-processing is implemented in MATLAB and run on a computer equipped with an Intel Xeon processor at 3.50 GHz (6 cores) CPU and 48 Gb of RAM. Currently, the processing time to produce 8 images of size 256 × 256 pixels (linear retardance (R_L_) and its azimuth (α_R_), linear diattenuation (D_L_) and its azimuth (α_D_), circular retardance (R_C_), circular diattenuation (D_C_), average depolarization (Δ) and the transmittance of the specimen (*m*_00_ element of the Mueller matrix)) is 10 s. Retardances *R*_*L*_ and *R*_*C*_ correspond to the phase anisotropy (optical path length) for respectively two orthogonal linear polarization states or two orthogonal circular polarization states. Retardance is measured here as a phase angle from 0° to 180°. Diattenuations *D*_*L*_ and *D*_*C*_ correspond to the amplitude anisotropy, defined as (*T*_*max*_ − *T*_*min*_)/(*T*_*max*_ + *T*_*min*_), where *T*_*max*_ and *T*_*min*_ are the maximum and minimum intensity transmittances for respectively two orthogonal linear polarization states or two orthogonal circular polarization states. Diattenuation is equal to 1 for a perfect polarizer (*T*_*min*_ = 0) and to 0 without anisotropy effects in amplitude. Azimuths α_*R*_ and α_*D*_ are measured from −90° to 90°. Finally, depolarization parameter Δ is equal to 0 for a non-depolarizing specimen and to 1 for a total depolarizer. Steps of post-processing are described as follows:

1. Fourier Transform of the channelled spectra leads to 12 peaks whose complex values are expressed as linear combinations of Mueller components, plus a peak at zero frequency, so 25 real values to determine the 16 Mueller components.
2. Matrix inversion to solve the system of linear equations permits to obtain the Mueller matrix *M*_*x*,*y*_ at each point on the sample. This matrix inversion depends on the thickness of the chromatically dispersed retarders present in the PSG and PSA blocks that can change by thermal expansion. A protocol detailed in ref.^31^ and based on two reference linear polarizers inserted on either side of the microscope, is used to calibrate the spectrally encoded Mueller microscope.
3. Mueller matrix is multiplied by the inverse of the Mueller matrix measured without the specimen in order to correct the polarimetric response of the microscope at each pixel of the sample.
4. Mueller matrix images are interpreted by applying the polar decomposition proposed by Lu and Chipman^24^, the last step being the most time-consuming (6 s). Other matrix decompositions^54,55^ exist which have proved their efficiency for strong depolarized samples characterized in reflection. However, for thin biological specimens imaged in transmission under microscope, scattering is not the main effect – except sample interfaces – and Lu and Chipman decomposition, which is the most widely used, has been chosen for this report.

The precision of the Mueller microscope through the Mueller analysis was evaluated using a spatial homogeneous linear retarder (zero-order half-wave plate at 830 nm) and a linear polarizer (ColorPol VIS-IR polarizer, Codixx) according to the field of view and the objectives used in the manuscript. Mean values and standard deviations (std) are given in the following form: mean value ± (std at the centre of the image, std over the image) after the Lu and Chipman decomposition. The standard deviation at the centre of the image has been calculated with 1000 successive channelled spectra in time, while it has been calculated over the image with 256 × 256 channelled spectra associated to the pixels of the image. The zero-order half-wave plate at 830 nm is a quartz plate whose theoretical retardance is 138.79° at 1060 nm and 139.06° for a broadband source centred at 1060 nm with a 90 nm-bandwidth. Table 1 shows the polarimetric values associated to the half-wave plate at 830 nm according to the objective and the field of view. The mean values of the linear retardance R_L_ are close to the theoretical value (maximum relative error of 0.5%) and vary slightly with the objective certainly due to the collection of light by the condenser lens. The standard deviation of R_L_ is equal to 0.1° at the centre of the image and increases up to 0.3° for a 90 × 90 μm field of view and 1° for a 1.7 × 1.7 mm field of view. Even if the polarimetric response of the microscope is numerically corrected at each point, it is not enough to get rid of it completely, particularly on the edges of the image for large fields of view. Thus retardance resolution can be estimated as being equal to 0.1° at each point but a retardance image must exhibit a contrast greater than 0.3° (or 1°) over the 90 × 90 μm field of view (or 1.7 × 1.7 mm) to be above the background. Moreover, due to the angular distribution of the wave vectors at the sample, the measured depolarization parameter Δ is slightly biased by the NA of the objective from Δ = 0.045 for NA = 0.16 (4X objective) to Δ = 0.078 for NA = 0.75 (20X objective).

**Table 1:** Polarimetric parameters measured for a zero-order half-wave plate at 830 nm.

Objectives	R_L_	R_C_	D_L_(×10^−3^)	D_C_(×10^−3^)	Δ (×10^−3^)
X4, 1.7 × 1.7 mm	138.4° ± (0.1°, 1°)	4° ± (0.2°, 3°)	14 ± (2, 4)	13 ± (2, 4)	45 ± (2, 7)
X20, 90 × 90 μm	139.1° ± (0.1°, 0.3°)	0.13° ± (0.1°, 0.1°)	6 ± (2, 3)	4 ± (2, 3)	78 ± (2, 4)

Values of linear retardance R_L_, circular retardance R_C_, linear diattenuation D_L_, circular diattenuation D_C_ and depolarization parameter Δ are given according to 4X (NA = 0.16) and 20X (NA = 0.75) objectives with corresponding 1.7 × 1.7 mm and 90 × 90 μm fields of view. Mean values and standard deviations (std) are given in the following form: mean value ± (std at the centre of the image, std over the image).

Table 2 shows the polarimetric values associated to the linear polarizer (10^5^:1 extinction ratio, ±20° acceptance angle) according to the objective and the field of view. Retardance parameters are not presented in Table 2 because noise propagation through Lu and Chipman decomposition does not permit to calculate them for nearly perfect diattenuators (close to singular matrices). The mean values of the linear diattenuation D_L_ are close to the theoretical value (maximum relative error of 1.1%) with a standard deviation equal to 0.005 at the centre of the image. However the standard deviation increases with the extension of the field of view from 0.005 for 90 × 90 μm image size to 0.013 for 1.7 × 1.7 mm. Lastly NA has an impact on the depolarization parameter Δ (Δ = 0.021 for NA = 0.16 and Δ = 0.048 for NA = 0.75) although the bias is less important due to the high acceptance angle of the linear polarizer used for the assessment of the Mueller microscope.

**Table 2:** Polarimetric parameters measured for a linear polarizer.

Objectives	D_L_(×10^−3^)	D_C_(×10^−3^)	Δ (×10^−3^)
X4, 1.7 × 1.7 mm	1000 ± (5, 13)	5 ± (3, 4)	21 ± (3, 7)
X20, 90 × 90 μm	989 ± (5, 5)	10 ± (5, 5)	48 ± (3, 8)

Values of linear diattenuation D_L_, circular diattenuation D_C_ and depolarization parameter Δ are given according to 4X (NA = 0.16) and 20X (NA = 0.75) objectives with corresponding 1.7 × 1.7 mm and 90 × 90 μm fields of view. Mean values and standard deviations (std) are given in the following form: mean value ± (std at the centre of the image, std over the image).

#### Potato starch granule specimen

Starch granules were isolated from a fresh cut of potato and placed on a microscope coverslip. Images have been produced within a few minutes after the cut.

#### Collagen specimen with acid

Aponeurosis tissue was harvested from the leg muscle of a raw chicken bought in a supermarket, transferred to a glass plate and frozen. The recording of images started one minute after removing from the freezer. Acid used in the paper is 37% hydrochloric acid.

#### Liver fibre specimen

The unstained liver specimen, provided by Bruno Turlin of the Department of Pathology (Pontchaillou hospital, Rennes, France), was obtained from a cohort of large surgical biopsies of human patients with chronic liver fibrosis, as previously described in the work published by one of the co-authors (Y. Le Grand^56^). The specimen consists in a 50 µm thick acute section from a surgical biopsy that had been embedded in paraffin and mounted between a glass slide and a coverslip. The liver histological status of the specimen was assessed by a trained pathologist using the Fibrosis-Metavir scoring system^57^ that ranges from F0 (no fibrosis) to F4 (cirrhosis). Briefly, fibrosis is staged on a scale from F0 to F4: F0 = no Fibrosis, F1 = fibrosis without septa, F2 = few septa, F3 = numerous septa without cirrhosis, and F4 = cirrhosis. The fibrosis stage for the specimen used in this paper is F4. Mueller and SHG microscopy was performed without dewaxing or staining the specimen. The cross-polarized image was acquired with a commercial microscope (Zeiss Axioskop 2) equipped with a 2.5X/0.075NA objective lens. All methods and protocols were performed in accordance with guidelines and regulations approved by the Regional University Hospital in Rennes, France. Informed consent was obtained from all patients in the original cohort for liver biopsies.

## Supplementary information

Supplementary Video S1 online

Supplementary Video S2 online

## Data Availability

The datasets generated and/or analysed in the current paper are available from the corresponding author on reasonable request.
